# Establishing a prognostic threshold for total copy number variation within adult *IDH*-mutant grade II/III astrocytomas

**DOI:** 10.1186/s40478-019-0778-3

**Published:** 2019-07-26

**Authors:** Kanish Mirchia, Matija Snuderl, Kristyn Galbraith, Kimmo J. Hatanpaa, Jamie M. Walker, Timothy E. Richardson

**Affiliations:** 1 Department of Pathology, State University of New York, Upstate Medical University, Syracuse, NY 13210 USA; 2 Department of Pathology, New York University Langone Health, New York City, NY 10016 USA; 3 Department of Pathology, University of Texas Southwestern Medical Center, Dallas, TX 75390 USA; 4 Department of Pathology, University of Texas Health Science Center, San Antonio, TX 78229 USA; 5 Glenn Biggs Institute for Alzheimer’s & Neurodegenerative Diseases, University of Texas Health Science Center, San Antonio, TX 78229 USA

**Keywords:** Copy number variation, CNV, Copy number alterations, CNA, IDH mutation, Astrocytoma, Glioma, Glioblastoma, GBM, TCGA

Prior to the 2016 WHO update, adult astrocytomas were graded exclusively by histologic features, where grade III anaplastic astrocytomas were separated from grade II diffuse infiltrating astrocytomas on the basis of mitotic figures, and glioblastomas (GBM; grade IV) were defined based on the presence of microvascular proliferation and/or tumor necrosis [9]. With the establishment of the 2016 WHO Classification of Tumours of the Central Nervous System guidelines, adult astrocytomas have been segregated into *IDH*-wildtype and *IDH*-mutant groups due to the significant prognostic advantage conferred by the presence of an *IDH1* or *IDH2* mutation [9, 14]. Since this update, much work has been done to establish additional prognostic factors in both *IDH*-wildtype and *IDH*-mutant astrocytomas to further subclassify these groups and to aid in the understanding of the underlying biology [1–3, 5].

We have previously investigated the influence of total copy number variation (CNV) on clinical outcome in adult astrocytomas in a variety of cohorts, including cases from The Cancer Genome Atlas (TCGA) dataset [10–13]. In *IDH*-mutant lower-grade gliomas (grades II and III), elevated total CNV (16–22%) is associated with poor clinical outcome (defined in these earlier reports as rapid progression to GBM and patient survival intervals < 24 months) compared to histologically similar tumors with lower total CNV (8–10%), and the level of total genomic CNV is inversely correlated with both progression-free and overall survival in linear regression models. Total CNV appears to have no prognostic value in *IDH*-wildtype astrocytoma or *IDH*-mutant GBM groups [10–12].

Unlike other prognostic factors, however, CNV is more difficult to utilize clinically, as it is not a simple “present-or-absent” model like *IDH1/2* mutations. To address this, we used the cBioPortal interface [4, 7] to reanalyze the survival and CNV data from *IDH*-mutant grade II/III astrocytomas in our previous publications (*n* = 67) [10, 12] to establish a simple and reasonable threshold that could be used to reliably predict clinical outcomes within the *IDH*-mutant, 1p/19q-retained lower-grade astrocytoma subgroup. We defined CNV as the percentage of the genome with alterations meeting the criteria of log2 > 0.3; copy number data was derived from Affymetrix SNP6 (Santa Clara, CA, USA) and Agilent 224 K/415 K (Santa Clara, CA, USA) platforms [1]. Preliminary data from our previous studies suggested potential clinically useful CNV cutoff levels of 10, 15, and 18% [10, 12].

Using Kaplan-Meier analysis on cases from our previous publications and additional cases from the TCGA database (total *n* = 194 *IDH*-mutant lower-grade astrocytomas), we evaluated each of these potential thresholds. There was a significant survival difference between cases at each threshold evaluated: 10% (< 10% CNV, 105.2 month median survival; > 10% CNV, 62.2 month median survival; *p* = 0.0020) (Fig. 1a), 15% (< 15% CNV, 105.2 month median survival; > 15% CNV, 50.1 month median survival; *p* < 0.0001) (Fig. 1b), and 18% (< 18% CNV, 98.2 month median survival; > 18% CNV, 41.2 month median survival; *p* = 0.0003) (Fig. 1c). There was no significant patient survival difference in the 10% vs 15% CNV thresholds, and an additional group with CNV between 10 and 15% (130.8 month median survival) was not significantly different than the group with < 10% CNV (*p* = 0.6003), however these cases had significantly better clinical outcomes than cases with > 15% CNV (*p* = 0.0113) (Fig. 1d), suggesting 15% as the better cutoff point. No significant difference was found between the 15 and 18% thresholds (Fig. 1e) or the 10 and 18% thresholds (Fig. 1f), however the 18% threshold would exclude > 50% of cases from one of our previously reported poorly performing cohorts [12]. The 15% CNV threshold had a sensitivity of 85%, specificity of 90%, positive predictive value (PPV) of 77%, and negative predictive value (NPV) of 94%. The 10% CNV threshold had a relatively low specificity (57%) and PPV (32%) and the 18% CNV threshold had a lower sensitivity (75%) (Table 1). Based on these models, we suggest that a judicious CNV cutoff for predicting poor clinical outcome in adult *IDH*-mutant lower-grade astrocytomas would be around 15%. It is important to note that these results are from a single, albeit relatively large, data source and as such should be validated prospectively. It would be of interest to evaluate our suggested cutoff using other large-scale cohorts to confirm our recommendation of 15% CNV or alternatively help improve the robustness of our model and refine this threshold.

**Fig. 1:**
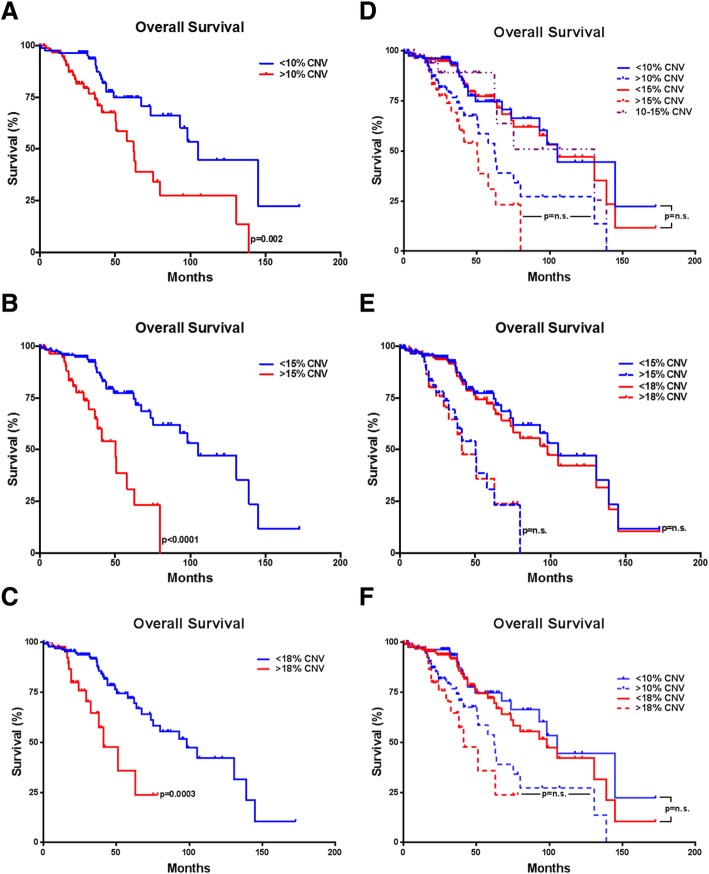
Kaplan-Meier survival curves demonstrating survival differences using 10% overall CNV as a threshold for poor clinical outcome (*p* = 0.0020) (**a**), using 15% overall CNV as a threshold for poor clinical outcome (*p* < 0.0001) (**b**), and using 18% overall as a threshold for poor clinical outcome (*p* = 0.0003) (**c**). Kaplan-Meier survival curves demonstrating no significant difference between 10 and 15% CNV thresholds (additionally, < 10% vs 10–15% *p* = 0.6003; 10–15% vs > 15% *p* = 0.0113) (**d**), no significant difference between 15 and 18% CNV thresholds (< 15% vs < 18, *p* = 0.5949; > 15% vs > 18% *p* = 0.9015) (**e**), and no significant difference between 10 and 18% CNV thresholds (< 10% vs < 18, *p* = 0.4791; > 10% vs > 18% *p* = 0.2672) (**f**). Copy number variation is expressed as a percentage of the total genome, log2 > 0.3 as reported previously [10, 12]

**Table 1:** Comparison of 10, 15, and 18% CNV thresholds

CNV Level	Sensitivity	Specificity	PPV	NPV
10%	88%	57%	32%	96%
15%	85%	90%	77%	94%
18%	75%	93%	75%	93%

While total CNV is not currently a regularly measured factor at the time of diagnosis, recent proof-of-concept studies have shown that genetic and epigenetic variables such as CNV and various mutations, individual gene amplifications/deletions, and chromosomal gains/losses can be identified rapidly [6, 8], and thus CNV estimates may soon be available within days of histologic diagnosis, raising the possibility for its use as an additional clinical factor guiding prognosis in *IDH*-mutant lower-grade astrocytomas.

## Data Availability

The full dataset used in this study is freely available at www.cbioportal.org and https://www.cancer.gov/about-nci/organization/ccg/research/structural-genomics/tcga.
